# Comparative genomics of brain size evolution

**DOI:** 10.3389/fnhum.2014.00345

**Published:** 2014-05-21

**Authors:** Wolfgang Enard

**Affiliations:** Department of Biology II, Ludwig Maximilian University MunichMunich, Germany

**Keywords:** brain size evolution, comparative method, comparative genomics, brain development, microcephaly

## Abstract

Which genetic changes took place during mammalian, primate and human evolution to build a larger brain? To answer this question, one has to correlate genetic changes with brain size changes across a phylogeny. Such a comparative genomics approach provides unique information to better understand brain evolution and brain development. However, its statistical power is limited for example due to the limited number of species, the presumably complex genetics of brain size evolution and the large search space of mammalian genomes. Hence, it is crucial to add functional information, for example by limiting the search space to genes and regulatory elements known to play a role in the relevant cell types during brain development. Similarly, it is crucial to experimentally follow up on hypotheses generated by such a comparative approach. Recent progress in understanding the molecular and cellular mechanisms of mammalian brain development, in genome sequencing and in genome editing, promises to make a close integration of evolutionary and experimental methods a fruitful approach to better understand the genetics of mammalian brain size evolution.

## A comparative approach for genotype-phenotype associations

Which genetic changes took place during mammalian, primate and human evolution to build a larger brain? To answer this question, one has to determine brain size and gene or genome sequences across species and reconstruct changes on the phylogeny of the species. This allows correlating genetic and phenotypic changes that occur independently on different lineages of the phylogeny. This comparative method has a long tradition in evolutionary biology for correlating two or more phenotypic traits across species (Felsenstein, 1985; Harvey and Pagel, 1991; Pagel, 1999; Freckleton, 2009) and methods specifically designed to correlate genetic substitutions rates with phenotypic traits have also recently been developed (O’Connor and Mundy, 2009, 2013; Lartillot and Poujol, 2011). Advances in DNA sequencing technologies have made the determination of gene and genome sequences much easier in recent years and will increase the number of high-quality mammalian genomes available for comparative analyses and phylogenetic inferences eventually to most mammal and vertebrate species. (Genome 10k Community of Scientists, 2009). While any piece of DNA containing tissue is in principle enough to determine the genomic sequence of a species, measuring phenotypic variables across a range of species can be practically much more difficult. Fortunately, due to decades of interest, fairly large data sets are available for brain and body size (Stephan et al., 1981; Boddy et al., 2012; Montgomery et al., 2013). Related parameters such as brain folding (Zilles et al., 2013; Lewitus et al., 2013b), neuronal numbers (Herculano-Houzel, 2011) and cortical thickness (Lewitus et al., 2013a) are also available for mammalian species, as well as correlated life history traits such as life span, gestation time or fertility (Isler and Van Schaik, 2012). In addition, the cellular and molecular understanding of brain development has advanced considerably (Fietz and Huttner, 2011; Lui et al., 2011; Sun and Hevner, 2014) and is increasingly studied in species other than the mouse (e.g., Fietz et al., 2010; Hansen et al., 2010; Reillo et al., 2011; Kelava et al., 2012; Betizeau et al., 2013). Hence, now might be a good time to evaluate the possibilities and challenges of approaching the genetics of brain size evolution using cross-species comparisons. While this perspective focuses on human, primate and mammalian brain size, the same principles will apply to the analysis of other heritable traits that vary across species (Enard, 2012).

## Examples of genes correlating with brain size changes

Changes in a phenotype such as brain size can principally be correlated with changes at any genetic level, from single positions, regulatory elements, promoters, protein domains, whole proteins to pathways. However, at what level genetic convergence most often occurs and hence at what level the signal to noise ratio is optimal is unknown (but see Stern, 2013). Either way, the amount of changes at putatively functional positions is expected to be larger when more time, i.e., more mutations occur on a lineage and hence need to be normalized to the amount of changes at putatively neutral positions, i.e., to the mutation rate. Most frequently this approach is applied to the evolution of protein coding genes by estimating the ratio of the nonsynonymous (i.e., amino acid changing) to the synonymous (i.e., silent) nucleotide substitution rates (often called dN/dS or ω). A dN/dS <1 for particular codons, domains or the whole protein is taken as evidence for positive selection. However, positive selection needs to act repeatedly either on the same site across species or on several sites along a protein to be reliably detected. Since most positions in a protein are usually conserved, i.e., evolve at a dN/dS <1, one has little power to detect positive selection acting on a few sites in a conserved protein using dN/dS <1 as a criterion. A less strict criterion is to use an elevated rate of dN/dS as an indicator of positive selection, if the alternative explanation of less negative selection, i.e., relaxed constraint, is unlikely (see e.g., Nielsen, 2005; Jensen et al., 2007) for a review on methods to detect natural selection).

These approaches have also been used to study the evolution of brain size associated genes, especially for genes involved in primary microcephaly, since this developmental disease affects primarily the size of the brain without major effects on neuronal migration or cortial folding (Kaindl et al., 2010; Gilmore and Walsh, 2013). While initial studies did find evidence for positive selection across some primate lineages for several genes (reviewed in Gilbert et al., 2005), the correlation with changes in brain size was usually not explicitly tested, impeding the interpretation of these findings (Woods et al., 2005; Montgomery et al., 2014).

Such an explicit correlation has been done by Montgomery et al. for the four microcephaly genes *ASPM*, *CDK5RAP2*, *CENPJ* and *MCPH1* across 21 anthropoid primates (Montgomery et al., 2011) and *NIN*, a centrosomal protein associated with asymmetric cell division (Montgomery and Mundy, 2012b). While they find strong evidence that all these genes are affected by positive selection, they find a significant correlation of dN/dS with absolute neonatal brain size only for *ASPM*, *CDK5RAP2* and *NIN*, but not for *CENPJ* and *MCPH1* or eight other “control” genes with available data from 10–20 anthropoid primates. Since the correlation of *CDK5RAP2* depends partly on the rate of dS and the correlation for *NIN* is largely restricted to catarrhines, the most convincing case is maybe *ASPM*, also because its dN/dS is additionally correlated with brain size reductions in callitrichids (Montgomery and Mundy, 2012a).

## Adding information by adding species

The found correlations are promising and the approach should be extended to more genes and eventually entire genomes. A genome-wide approach would allow to gauge how exceptional the findings for the studied microcephaly genes are and it would allow to correct for relevant genome-wide effects such as differences in evolutionary rates due to differences in population size across lineages. Since selection is stronger in larger populations (see e.g., Lanfear et al., 2014 for a recent review), controlling for this effect on measures like dN/dS could increase the sensitivity and specificity of the approach and potentially explain some of the outliers found by Montgomery *et al*. Further work is necessary to choose the most powerful comparative method (e.g., Lartillot and Poujol, 2011; O’Connor and Mundy, 2013), that ideally integrates within-species variation (Felsenstein, 2008 #424) and systematically analyses the influence of variables such as body size, adult brain size, cortical folding (Lewitus et al., 2013b; Zilles et al., 2013), neuronal numbers (Herculano-Houzel, 2011) or cortical thickness (Lewitus et al., 2013a).

Adding more species to an analysis is certainly another way to improve the approach, in particular if this adds independent variation in brain size to a phylogeny. However, if additional species, e.g., from different mammalian orders or even additional vertebrate classes differ considerably in the developmental and/or genetic mechanisms, they could also add too much noise to the analysis. In addition, the alignment of orthologous genomic elements becomes increasingly difficult with increasing phylogenetic distances, in particular when including different classes of vertebrates (see e.g., Cooper and Brown, 2008) for a discussion on the phylogenetic scope in a functional context).

To what extent brain development across mammals is actually different, is just beginning to be explored in more detail. One piece of evidence that it is not identical comes from the different scaling of brain size and neuronal numbers e.g., in primates and rodents (Herculano-Houzel, 2011). Also the finding that the knockout of genes resulting in severe reductions in brain size in humans, just has mild effects in mice (e.g., Pulvers et al., 2010) and that expanding basal progenitors results in different brain size increases in mice and ferrets (Nonaka-Kinoshita et al., 2013) indicates that considerable differences might exist among mammals.

Until recently, it was also thought that brain development in primates might be generally different from other mammals due to the presence of an outer subventricular zone (OSVZ; Smart et al., 2002; Kriegstein et al., 2006) that contains neural progenitors—called basal radial glia (bRGs)—that give rise to the majority of neurons in the folded (gyrencephalic) primate cortex (Hansen et al., 2010). However, it turned out that an OSVZ and bRGs are actually not primate-specific since they are also found in the ferret, a gyrencephalic carnivore (Fietz et al., 2010; Reillo et al., 2011), in the agouti, a gyrencephalic rodent (García-Moreno et al., 2012) and at low abundance also in the unfolded (lissencephalic) cortex of mice (Wang et al., 2011). Although bRGs are probably necessary to build a large, folded cortex, they might not be sufficient, since also the marmoset, a lissencephalic primate, contains many bRGs (García-Moreno et al., 2012; Kelava et al., 2012). In fact, a gyrencephalic cortex and hence potentially also bRGs, might have been present already in the mammalian ancestor (Lewitus et al., 2013b; Romiguier et al., 2013). These findings argue that brain development might actually be more similar across mammals than previously thought and that primates are mechanistically not special or “advanced” in this respect. It is interesting that the primate-specific occurrence of an OSVZ was apparently a plausible hypothesis for almost 10 years, solely based on its presence in monkeys and humans and on its absence in mouse (see Rigato and Minelli, 2013) for an analysis on the prevalence of progressionist terms in the current scientific literature).

Although these comparative developmental studies are practically challenging, they will hopefully be extended to better understand similarities and differences in brain development across mammals. This should eventually improve the basis for choosing the proper phylogenetic scope for the genomic analyses, potentially including also other vertebrates. Currently, it seems a reasonable compromise for genomic studies to include anthropoid primates given their close relationship to humans and their well-studied variations in brain size, include rodents, given the central role of the mouse as model organism for brain development and include carnivores such as the ferret that emerges as a model organism to experimentally study gyrencephalic brains (e.g., Nonaka-Kinoshita et al., 2013). Cetaceans (whales and dolphins) might be another relevant mammalian group to include, given their independent evolution of big brains. However, the relationship of brain and body size evolution might be different and more complex compared to terrestrial mammals (Montgomery et al., 2013) and since their brain development is difficult to study for practical reasons, it is unclear how similar it is to other mammals. A recent study claimed that *ASPM* evolution correlates with brain size changes also in cetaceans (Xu et al., 2012), which would strongly suggest to include this group in future genotype-phenotype associations. Unfortunately, this claim is statistically not well supported as a recent reanalysis suggests (Montgomery et al., 2014). Eventually all mammalian genomes will be available and one will hopefully be able to gauge from the data, how similar the genetic basis for brain development and evolution is across mammalian groups.

However, even if hundreds of species could be included in such an analysis, it is clear that the information is limited due to the limited number of independent changes of brain size in the phylogeny. So, the power of genotype-phenotype correlations across species is *per se* limited and genome-wide significance is unlikely to be reached for many genes or genetic elements. However, power can be increased if one adds information, for example by prioritizing or weighing genes and genetic elements based on their relevance for brain size development, e.g., in a model species like the mouse. One way to view this is that one needs to understand the cellular and molecular basis of brain size development to reduce the genomic search space for a comparative approach.

## Adding information by reducing the genomic search space

The understanding of the cellular and molecular processes underlying brain development is currently progressing at an exciting speed. It is beyond the scope of this perspective to summarize the field (see e.g., Lui et al., 2011; Fietz and Huttner, 2011 or Sun and Hevner, 2014 for reviews), but a few recent findings might be of particular importance (see also Ghosh and Jessberger, 2013). Recent experimental (Nonaka-Kinoshita et al., 2013) and theoretical (Lewitus and Kalinka, 2013) modeling now strongly suggests that an expansion of basal progenitors (bRGs) can lead to a larger and more folded cortex of similar thickness as it is generally observed across mammalian brain size expansions. Although bRGs turn out to be quite heterogenous (Betizeau et al., 2013; Pilz et al., 2013), it seems likely that this cell type or subsets of this cell type play a crucial role in mammalian brain size evolution.

First molecular players like *Trnp1* (Stahl et al., 2013) or *Smarcc2* (BAF170) (Tuoc et al., 2013) have been identified that when experimentally down-regulated or up-regulated caused an increase or decrease, respectively, in cortical size. Remarkably, in the case of *Trnp1* it could be shown that experimentally reducing *Trnp1* expression levels leads to an increase of bRGs and cortical folding in the mouse (Stahl et al., 2013). These results might make *Trnp1* currently the best candidate gene for being directly involved in regulating mammalian brain size, but its sequence evolution has not yet been correlated with brain size evolution.

While a correlation of their protein coding sequence in the described comparative framework is now already possible, the location of the involved regulatory regions, in particular the distantly located enhancers, is lacking. Fortunately, methods such as DNAse-Seq and ChIP-Seq to identify and analyze such regions on a genome-wide scale have massively increased in recent years (e.g., Thurman et al., 2012). These regulatory regions are fairly cell-type specific (e.g., Neph et al., 2012), which is the reason why they are considered good targets for evolutionary change (e.g., Carroll, 2008) and also the reason why it would be important to determine them in the very cell type in question. So extending approaches (Fietz et al., 2012) to catalog the genes expressed in bRGs and their regulatory regions might be an important next step for an evolutionary analysis of the existing and putative future candidate genes. Ideally this should be done in as many species as possible, but will for practical reasons probably be limited to a few species such as mouse, human and potentially macaque, marmoset or ferret.

## Experimentally following up hypotheses

How strong the resulting correlations of genetic changes in genes or genetic elements and changes in brain size will be is currently unclear. If few genetic changes in many different elements did occur during brain size evolution, the information content will be fairly low. However, convergence at the genetic level might be more frequent than initially thought, as an increasing number of cases show (Stern, 2013). Nevertheless, the comparative approach will probably only rarely provide strong associations with great confidence and more often generate hypotheses that need to be followed up experimentally. Consequently, it will depend on the validity and efficiency of experimental assays whether the outlined comparative genomics approach will be eventually be fruitful. It is beyond the scope of this perspective to lay out the different possibilities, in particular since these differ considerably e.g., if the hypothesis is about few genetic changes in a short stretch of DNA sequence that can be changed in one step in an organism by genome engineering or if the hypothesis is about several changes spread out over an entire gene or even a set of genes. Furthermore, possibilities will differ whether these changes can be tested in cell culture, in cells in a developing brain or must be studied in an entire organism. The mouse will probably continue to be the major model organism for studying brain development for practical reasons, although it is could be a major drawback that the mouse contains only very few bRGs, crucial progenitor cells for brain size evolution (see above). Hence, it might be important to relate phenotypes in the mouse to species with a folded cortex, like recently done with the ferret (Nonaka-Kinoshita et al., 2013). The only study that I am aware of that has so far directly functionally tested an evolutionary hypothesis for brain size evolution is from Pulvers et al. (2010) where it was shown that a mouse transgenic for a Bacterial artificial chromosome (BAC) containing the human ASPM can qualitatively rescue a mouse ASPM knockout. However, a more quantitative comparison involving ASPM alleles from additional species like the chimpanzee is lacking so far and would be interesting, given that ASPM is the gene for which the best correlation between protein evolution and brain size changes has been seen so far (see above). Another exciting prospect, in particular for evolutionary questions, is to model at least some aspects of brain development using stem cells and a recent landmark paper suggests that this might be possible (Lancaster et al., 2013). This would allow researchers to access brain developmental stages e.g., for a range of primates (Enard, 2012; Marchetto et al., 2013a, b; Wunderlich et al., 2014) and together with the increasing possibilities for genome engineering (Gaj et al., 2013) could be a major platform to test alleles from different species in different genomic backgrounds.

## Conclusion

In conclusion, knowledge about brain development as well as genomic and phenotypic information might have reached a critical mass to leverage comparative genomic data to inform brain evolution and development. To this end it will be important to optimize statistical methods, improve knowledge on the evolution of brain size changes, annotate the genome with gene expression and chromatin states from relevant cell types like the bRGs at least in one mammalian species and explore possibilities to test resulting hypotheses in experimental systems. The unique type of information present in comparative data should be worth the effort.

## Conflict of interest statement

The author declares that the research was conducted in the absence of any commercial or financial relationships that could be construed as a potential conflict of interest.
